# A myzozoan-specific protein is an essential membrane-anchoring component of the succinate dehydrogenase complex in *Toxoplasma* parasites

**DOI:** 10.1098/rsob.230463

**Published:** 2024-06-05

**Authors:** Soraya M. Zwahlen, Jenni A. Hayward, Capella S. Maguire, Alex R. Qin, Giel G. van Dooren

**Affiliations:** ^1^ Research School of Biology, Australian National University, Canberra, Australian Capital Territory, Australia

**Keywords:** *Toxoplasma*, apicomplexa, mitochondria, electron transport chain, tricarboxylic acid cycle

## Abstract

Succinate dehydrogenase (SDH) is a protein complex that functions in the tricarboxylic acid cycle and the electron transport chain of mitochondria. In most eukaryotes, SDH is highly conserved and comprises the following four subunits: SdhA and SdhB form the catalytic core of the complex, while SdhC and SdhD anchor the complex in the membrane. *Toxoplasma gondii* is an apicomplexan parasite that infects one-third of humans worldwide. The genome of *T. gondii* encodes homologues of the catalytic subunits SdhA and SdhB, although the physiological role of the SDH complex in the parasite and the identity of the membrane-anchoring subunits are poorly understood. Here, we show that the SDH complex contributes to optimal proliferation and O_2_ consumption in the disease-causing tachyzoite stage of the *T. gondii* life cycle. We characterize a small membrane-bound subunit of the SDH complex called mitochondrial protein ookinete developmental defect (MPODD), which is conserved among myzozoans, a phylogenetic grouping that incorporates apicomplexan parasites and their closest free-living relatives. We demonstrate that *Tg*MPODD is essential for SDH activity and plays a key role in attaching the *Tg*SdhA and *Tg*SdhB proteins to the membrane anchor of the complex. Our findings highlight a unique and important feature of mitochondrial energy metabolism in apicomplexan parasites and their relatives.

## 1. Introduction

Mitochondrial energy metabolism, which consists of processes like the tricarboxylic acid (TCA) cycle and the electron transport chain (ETC), is conserved throughout eukaryotic evolution and is important for the survival of many organisms [1,2]. Apicomplexans are a eukaryotic phylum of intracellular parasites that inflict a major burden on human societies, both through impacting human health and the health of important livestock. Apicomplexans include *Plasmodium* species, the causative agents of malaria, and *Toxoplasma gondii*, a ubiquitous parasite of humans and livestock that causes the disease toxoplasmosis [3,4]. Apicomplexans belong to a group of single-celled eukaryotes called the myzozoans, which also include chrompodellids and dinoflagellates [5,6]. Considerable evidence indicates that the mitochondrial energy metabolism of myzozoans has diverged considerably from well-studied organisms such as animals and yeast [7–9]. For example, although they contain canonical ETC complexes such as the cytochrome *bc*_1_ and cytochrome *c* oxidase complexes, many of the proteins that comprise these complexes are unique to myzozoans [8–12]. The essential function of these complexes has made the ETC a prime drug target in disease-causing apicomplexans [13–16].

The mitochondria of many myzozoans, including those of *T. gondii* and *Plasmodium falciparum*, harbour a functional TCA cycle. Like with the ETC, some of the enzymes that comprise this pathway have diverged considerably from the equivalent enzymes in animals [7,8,17–19]. The TCA cycle is dispensable for the disease-causing erythrocytic stage of the *P. falciparum* life cycle, probably reflecting the reliance of these parasites on ATP derived from glycolysis [20,21]. However, the TCA cycle becomes essential following the transmission of these parasites into the insect stage of the life cycle [20]. The importance of the TCA cycle in the disease-causing tachyzoite stage of *T. gondii* is less clear. Treatment with the aconitase inhibitor sodium fluoroacetate impairs the flux of carbons through the TCA cycle and inhibits parasite proliferation [18]. By contrast, depletion of the succinyl CoA synthetase enzyme of the TCA cycle, which catalyses the synthesis of succinate from succinyl-CoA, leads to an apparently mild defect in parasite proliferation [22], with its non-essentiality proposed to result from a γ-aminobutyric acid (GABA) shunt that enables this step of the TCA cycle to be bypassed [18].

Succinate dehydrogenase (SDH), or complex II of the ETC, is a protein complex that participates in both the TCA cycle and the ETC. It catalyses the oxidation of succinate to fumarate in the TCA cycle and transfers the resulting electrons to the oxidized form of coenzyme Q (ubiquinone) in the ETC. In animals, fungi and bacteria, the protein complex typically comprises the following four subunits: SdhA and SdhB form the matrix-localized, catalytic core of the complex, while SdhC and SdhD anchor the complex in the mitochondrial membrane and facilitate electron transfer to coenzyme Q [23]. SdhA is a large flavoprotein that catalyses the oxidation of succinate to fumarate in the TCA cycle, transferring electrons to a covalently attached flavin adenine nucleotide (FAD), forming FADH_2_ [23,24]. Electrons from FADH_2_ are transferred via three iron–sulfur clusters coordinated at SdhB to coenzyme Q. SdhB is bound to the transmembrane proteins SdhC and SdhD, with the high-affinity coenzyme Q binding site of the complex comprising residues from each of these proteins [25]. The reduced form of coenzyme Q then shuttles the electrons to complex III in the ETC from where they are donated to the terminal oxidase, complex IV, where O_2_ is reduced [1].

The catalytic SdhA and SdhB proteins of the SDH complex are highly conserved in eukaryotes and prokaryotes, while the membrane-spanning proteins are more divergent. In plants and trypanosomes, the SdhC and SdhD proteins have diverged considerably from equivalent subunits in other organisms, and extra subunits found in the SDH complex may contribute to the functions of the membrane component of the complex [26–28]. While the *T. gondii* genome encodes homologues of the SdhA and SdhB proteins, it lacks clear homologues of the membrane subunits [9,29]. This poses the question of what could be anchoring the *T. gondii* complex in the mitochondrial membrane. A recent ‘complexome’-based proteomic analysis of mitochondrial protein complexes from *T. gondii* parasites identified seven previously uncharacterized proteins that co-purified with the *Tg*SdhA and *Tg*SdhB proteins [12], although none were characterized further. A similar complexome study from *P. falciparum* demonstrated co-migration of homologues of several of the candidate SDH complex proteins identified in *T. gondii* with *Pf*SdhA and *Pf*SdhB [10,30]. One of the candidate SDH complex proteins was previously identified as a myzozoan-specific, mitochondrial protein in the rodent malaria *Plasmodium berghei* [31]. This protein was found to be essential for parasites to develop into the ookinete form during the mosquito stage of the parasite life cycle and therefore termed ‘mitochondrial protein ookinete developmental defect’ (MPODD) [31]. This mirrors the essentiality of the canonical SDH complex protein SdhA for ookinete development in *Plasmodium* parasites and the increased importance of mitochondrial energy metabolism in insect stages of these parasites [20,32,33].

In this study, we set out to characterize the importance of the SDH complex for the disease-causing tachyzoite stage of *T. gondii* parasites and explore its role in mitochondrial physiology. We demonstrate that the SDH complex is important but not essential for parasite proliferation and ETC activity *in vitro*. We further demonstrate that the *T. gondii* homologue of MPODD (*Tg*MPODD) is a *bona fide* member of the membrane component of the SDH complex, important for enabling the attachment of the catalytic SdhA and SdhB subunits to the membrane subunits of the complex, and therefore essential for SDH enzyme activity. Together, our data provide functional insights into a divergent and important mitochondrial protein complex in *T. gondii* parasites.

## 2. Results

### 2.1. *Tg*SdhB is an essential component of the SDH complex in *T. gondii*

**Figure 1.**
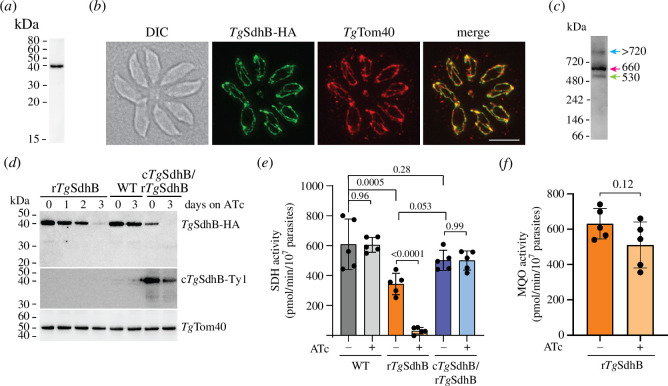
*Tg*SdhB is essential for SDH activity. (*a*) Western blot of proteins extracted from *Tg*SdhB-HA parasites separated by SDS–PAGE and detected with anti-HA antibodies. (*b*) Immunofluorescence assay of an eight-cell vacuole of *Tg*SdhB-HA parasites, probed with anti-HA antibodies to detect *Tg*SdhB-HA (green) and anti-*Tg*Tom40 antibodies to detect the mitochondrion (red), with the overlap shown in the merged image. DIC, differential interference contrast transmission image. Scale bar is 5 µm. (*c*) Western blot of proteins extracted from *Tg*SdhB-HA parasites in 1% (w/v) digitonin, separated by BN-PAGE and detected with anti-HA antibodies. Complex masses indicated with different coloured arrows were determined using relative migration distance (Rf) plots. (*d*) Western blot of proteins extracted from r*Tg*SdhB-HA parasites, WT parasites (*Tg*SdhB-HA) and the complemented line (c*Tg*SdhB-Ty1/r*Tg*SdhB-HA), cultured in the absence of ATc or the presence of ATc for the times indicated, separated by SDS–PAGE and detected using anti-HA (top), anti-Ty1 (middle) and anti-*Tg*Tom40 (bottom) antibodies. (*e*) SDH enzyme activity assays performed with WT parasites (grey), r*Tg*SdhB parasites (orange) and c*Tg*SdhB-Ty1/r*Tg*SdhB-HA parasites (blue) cultured in the absence or presence of ATc for 3 days. Column graphs show the mean SDH activity obtained from five independent experiments (individual points shown), with error bars representing standard deviation (s.d.). ANOVA followed by Tukey’s multiple pairwise comparisons test was performed, with relevant *p*-values shown. (*f*) Malate:quinone oxidoreductase (MQO) enzyme activity assays performed with r*Tg*SdhB parasites cultured in the absence or presence of ATc for 3 days. Column graphs show the mean MQO activity obtained from five independent experiments (individual points shown) taken from the same parasite extracts as (*e*), with error bars representing s.d. An unpaired two-tailed *t*‐test was performed comparing the minus and plus ATc conditions, with the *p*-value shown.

To begin to explore the role of the SDH complex in *T. gondii* biology, we set out to characterize *Tg*SdhB, the Fe–S protein of the complex. To facilitate its characterization, we introduced a haemagglutinin (HA) epitope tag at the 3′ end of the *Tg*SdhB open reading frame of *T. gondii*, creating a parasite line termed *Tg*SdhB-HA. SDS–PAGE western blotting and immunofluorescence assays of the HA-tagged protein revealed that, as described previously [12,34], *Tg*SdhB exists as a single species of ~40 kDa (figure 1*a*) and localizes to the mitochondrion (figure 1*b*). To test whether *Tg*SdhB exists in a protein complex, we extracted proteins from *Tg*SdhB-HA parasites in the mild detergent digitonin, separated them by BN-PAGE and then performed western blotting. We found that *Tg*SdhB is present in a main complex of ~660 kDa (figure 1*c*, magenta arrow), similar in size to the *Tg*SdhB-containing complex reported previously [12]. Additionally, we observed *Tg*SdhB in two less abundant complexes of ~530 kDa and >720 kDa (figure 1*c*, green and blue arrows, respectively). We conclude that *Tg*SdhB is a component of protein complexes in the mitochondrion.

Next, we wanted to explore the importance of *Tg*SdhB and the SDH complex generally, in parasite biology. We replaced the native promoter of *Tg*SdhB with an ATc-regulated promoter in the *Tg*SdhB-HA parasite line wherein another candidate SDH protein, *Tg*Sdh18 [12], had been tagged with a FLAG epitope (electronic supplementary material, figure S1). We termed the resulting parasite line ‘regulatable (r)*Tg*SdhB-HA/*Tg*Sdh18-FLAG’ (hereafter referred to as ‘r*Tg*SdhB’). To measure the extent of *Tg*SdhB knockdown upon the addition of ATc, we cultured r*Tg*SdhB parasites for 0–3 days on ATc, extracted parasite proteins, separated them by SDS–PAGE and performed western blotting. This revealed that *Tg*SdhB abundance was reduced in the r*Tg*SdhB line after 2 days on ATc and barely detectable after 3 days (figure 1*d*). By contrast, addition of ATc to the parental *Tg*SdhB-HA parasite line (hereafter referred to as wild type, WT) did not result in an appreciable change in *Tg*SdhB abundance after 3 days on ATc (figure 1*d*).

We investigated the importance of *Tg*SdhB for SDH enzyme activity using an absorbance-based enzymatic assay. In comparison to WT parasites, r*Tg*SdhB parasites cultured in the absence of ATc had approximately twofold lower SDH activity (figure 1*e*), possibly due to differences in the timing of expression of *Tg*SdhB from the ATc-regulatable promotor compared to the native promoter. Notably, we found that SDH activity decreased by 86% upon knockdown of *Tg*SdhB by the addition of ATc for 3 days, while SDH activity in the WT parasite line was unaffected by the addition of ATc (figure 1*e*). We also measured the activity of malate:quinone oxidoreductase (MQO), an unrelated enzyme that mediates the oxidation of malate in the TCA cycle and contributes electrons to the ETC [1], in r*Tg*SdhB parasites cultured in the absence or presence of ATc. We observed no significant change in MQO activity upon *Tg*SdhB knockdown (figure 1*f*), implying that the loss of SDH activity associated with *Tg*SdhB depletion is not the result of general impairment in TCA cycle or mitochondrial functions.

We complemented r*Tg*SdhB parasites with Ty1-tagged *Tg*SdhB expressed constitutively from the α-tubulin promoter, generating a line that we termed c*Tg*SdhB/r*Tg*SdhB. We extracted proteins from these parasites, separated them by SDS–PAGE and performed western blotting to demonstrate that the resulting *Tg*SdhB-Ty1 protein was expressed (figure 1*d*). We cultured c*Tg*SdhB/r*Tg*SdhB in the absence or presence of ATc for 3 days and measured SDH activity. We found that SDH activity in the complemented strain was unchanged upon the addition of ATc, and indistinguishable from SDH activity in WT parasites (figure 1*e*). Together, these experiments indicate that *Tg*SdhB-HA is critical for SDH activity in *T. gondii* parasites.

### 2.2. SDH is important but not essential for parasite proliferation and ETC activity in *T. gondii*

**Figure 2.**
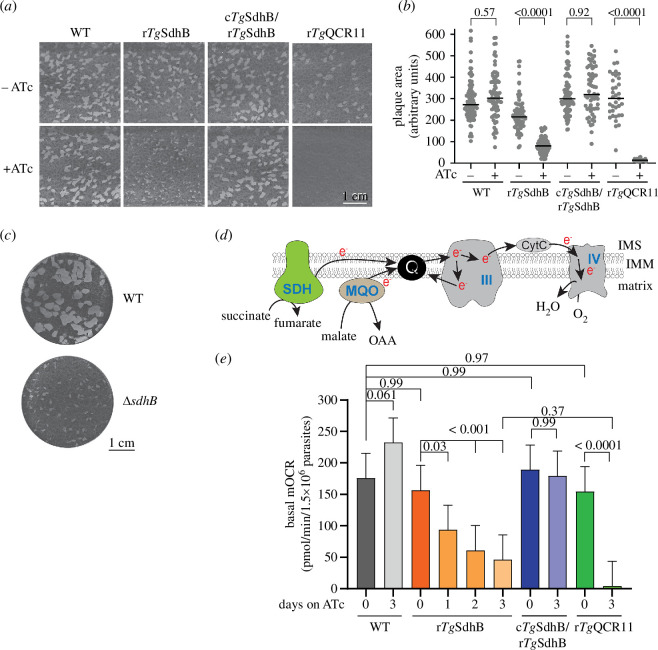
SDH is important for *T. gondii* proliferation and mitochondrial oxygen consumption. (*a*) Plaque assays with WT parasites (the *Tg*SdhB-HA parental line), r*Tg*SdhB-HA parasites, complemented c*Tg*SdhB-Ty1/r*Tg*SdhB-HA parasites and regulatable complex III mutant parasites (r*Tg*QCR11-FLAG) cultured in the absence (top) or presence (bottom) of ATc for 7 days. Images are from a single experiment and are representative of three independent experiments. (*b*) Quantification of plaque sizes from the experiment shown in (*a*). Black lines depict the median plaque size (in arbitrary units of area) for each parasite line and condition. A one-way ANOVA followed by Tukey’s multiple pairwise comparisons test was performed, with relevant *p* values shown. (*c*) Plaque assays comparing proliferation of ∆*sdhB* parasites to the corresponding WT line (Cas9-expressing RH strain parasites). Images are from a single experiment and are representative of two independent experiments. (*d*) Simplified schematic of the ETC of *T. gondii* parasites, showing the reactions relevant for this study. Electrons from the catabolism of carbon substrates by a range of mitochondrial dehydrogenases (including succinate dehydrogenase (SDH) and malate:quinone oxidoreductase (MQO)) results in the transfer of electrons (e^−^) to the mitochondrial inner membrane (IMM) electron carrier coenzyme Q (Q). Electrons are then donated via complex III and cytochrome *c* (CytC) to ultimately reduce O_2_ at complex IV. IMS, intermembrane space; OAA, oxaloacetate. (*e*) Basal mitochondrial oxygen consumption rate (mOCR) of WT (grey), r*Tg*SdhB-HA (orange), c*Tg*SdhB-Ty1/r*Tg*SdhB-HA (blue) and r*Tg*QCR11-FLAG (green) parasites cultured in the absence of ATc or presence of ATc for 1–3 days. A linear mixed-effects model was fitted to the data, with values depicting the least squares mean ± 95% confidence interval (CI) from three independent experiments. ANOVA followed by Tukey’s multiple pairwise comparisons test was performed, with relevant *p*-values shown.

Having established the importance of *Tg*SdhB for SDH activity, we next set out to determine the role and importance of SDH in the biology of the disease-causing tachyzoite stage of *T. gondii* parasites. We first investigated the contribution of *Tg*SdhB, and by extension the SDH complex, to overall parasite proliferation. We cultured WT and r*Tg*SdhB parasites with or without ATc for 7 days and compared sizes of plaques (zones of clearance in the host cell monolayer created by proliferating parasites). Plaque sizes decreased significantly in r*Tg*SdhB but not in WT parasites cultured in the presence of ATc (figure 2*a,b*), indicating that r*Tg*SdhB parasites cultured in the presence of ATc exhibit reduced proliferation. Complementation with constitutively expressed *Tg*SdhB (c*Tg*SdhB/r*Tg*SdhB) restored parasite proliferation in the presence of ATc (figure 2*a,b*). We next compared the importance of SDH for parasite proliferation with other components of the ETC. We performed plaque assays using a parasite line termed r*Tg*QCR11 wherein we could knockdown expression of *Tg*QCR11, a key protein of complex III, by the addition of ATc [11]. We found that plaque sizes were considerably smaller upon *Tg*QCR11 knockdown than upon knockdown of *Tg*SdhB (figure 2*a,b*).

Although these data indicate that *Tg*SdhB is less important for proliferation than complex III of the ETC, it is conceivable that the reduced proliferation defect we observe in the r*Tg*SdhB strain upon ATc addition is because of incomplete knockdown of *Tg*SdhB. To further test the essentiality of *Tg*SdhB for parasite proliferation *in vitro*, we therefore attempted to generate a *Tg*SdhB knockout. We integrated a phleomycin-resistance cassette into the open reading frame of the *Tg*SdhB genomic locus in a Cas9-expressing strain of *T. gondii* (electronic supplementary material, figure S2*a,b*). The resulting strain is expected to harbour a functional knockout of the *Tg*SdhB gene, and we therefore termed it ∆*sdhB*. We validated that ∆*sdhB* parasites were defective in SDH activity but not MQO activity via an enzymatic assay (electronic supplementary material, figure S2*c,d*). To test the effects of *Tg*SdhB knockout on parasite proliferation, we undertook a plaque assay. We found that *Tg*SdhB parasites were impaired in proliferation compared to the corresponding parental parasite strain but that plaques were still visible (figure 2*c*), similar to the moderate defect in proliferation observed in the r*Tg*SdhB line cultured in the presence of ATc. Taken together, these data indicate that although it is not essential, *Tg*SdhB, and by extension, the SDH complex, is important for optimal parasite proliferation *in vitro*.

Given the role of SDH in the mitochondrial ETC in other eukaryotes, we next explored the importance of *Tg*SdhB and the SDH complex for parasite respiration. We measured the effects of *Tg*SdhB knockdown on the basal mitochondrial O_2_ consumption rate (mOCR) in the parasite, using a previously established Seahorse XFe96 Flux analyser assay [9,11,35]. In this assay, we incubate parasites in medium containing the carbon sources glucose and glutamine. The resulting metabolism of these carbon substrates by the parasite, including by reactions in the TCA cycle, results in the transfer of electrons to coenzyme Q in the inner mitochondrial membrane (figure 2*d*). Electrons then transport via complexes III and IV and are ultimately donated to molecular oxygen, the final electron acceptor in the ETC. The rate at which oxygen is consumed by the parasite, therefore, correlates to ETC activity (figure 2*d*). We cultured WT, r*Tg*SdhB, r*Tg*SdhB/c*Tg*SdhB or r*Tg*QCR11 parasites in the absence of ATc or the presence of ATc for 1–3 days and then measured basal mOCR. We observed that basal mOCR decreased upon the knockdown of *Tg*SdhB, depleting by ~70% 3 days after the addition of ATc (figure 2*e*). In contrast, the addition of ATc had no effect on mOCR in either WT parasites or the complemented line (c*Tg*SdhB/r*Tg*SdhB) (figure 2*e*). Notably, knockdown of *Tg*QCR11 resulted in a more severe depletion of basal mOCR than knockdown of *Tg*SdhB (figure 2*e*), reminiscent of the more severe proliferation defect observed upon *Tg*QCR11 knockdown (figure 2*a,b*).

Taken together, our data indicate that the SDH complex from *T. gondii* contributes to parasite proliferation and mOCR but is less important for these processes than complex III of the ETC.

### 2.3. *Tg*MPODD is a myzozoan-specific component of the SDH complex of *T. gondii*

**Figure 3.**
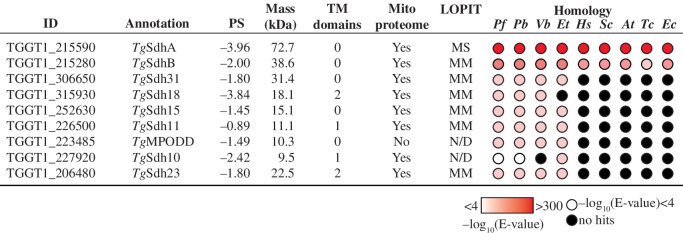
Candidate subunits of the SDH complex in *T. gondii* parasites.Information on the candidate SDH complex proteins identified in a recent complexome analysis of *T. gondii* mitochondria [12]; the table is modelled after [11,36]. ID: ToxoDB gene identification number [37]. Annotation: the name of the listed protein used in this manuscript. PS: phenotype score, a prediction of the importance of the listed gene for parasite proliferation, with fitness conferring genes typically having phenotype scores of <−2 [38]. Mass: the predicted molecular mass of the listed protein. TM domains: the predicted number of TMDs in the listed protein, as determined by CCTOP [39]. Mito proteome: detection of the listed protein in the mitochondrial proteome of *T. gondii* [9]. LOPIT: predicted subcellular localization of the listed protein in a spatial proteomic analysis of the T. gondii proteome using a localization of organelle proteins by isotope tagging approach [40]. MM, mitochondrial membranes; MS, mitochondrial soluble; N/D, not determined. Homology: the presence of homologues of the listed gene in the genomes of *Plasmodium falciparum* (*Pf*), *Plasmodium berghei* (*Pb*), *Vitrella brassicaformis* (*Vb*), *Eimeria tenella* (*Et*), *Homo sapiens* (*Hs*), *Saccharomyces cerevisiae* (*Sc*), *Arabidopsis thaliana* (*At*), *Trypanosoma cruzi* (*Tc*) and *Escherichia coli* (*Ec*). Homology searches were performed using HMMER for all species [41], with the exception of *Eimeria tenella*, which was searched using BLAST on the www.toxodb.org website [37]. Expected values (*E*-values) are indicated by coloured circles, with the intensity of red shading increasing in proportion with the −log_10_(*E*-value). Black circles indicate the absence of a detectable homologue in the target species.

The genome of *T. gondii* encodes homologues of the soluble, matrix-localized SdhA and SdhB subunits of SDH, but lacks clear homologues of the membrane subunits SdhC and SdhD. This raises the following question: what is anchoring the SDH complex in the mitochondrial membrane and facilitating electron transfer to ubiquinone? A recent complexome-based proteomic analysis of ETC complexes in *T. gondii* identified seven putative subunits of SDH that are restricted to apicomplexans and their nearest relatives such as chrompodellids (chromerids and colpodellids) and dinoflagellates, a group of organisms collectively referred to as myzozoans (figure 3) [12,30]. Six of these seven novel putative subunits were identified in an analysis of the mitochondrial proteome of *T. gondii* [9] and five were detected in the mitochondrial membrane fraction in a localization of organelle proteins by isotope tagging (LOPIT) approach (figure 3) [40]. Homologues of several of the candidate *T. gondii* SDH proteins were identified as candidate SDH complex proteins in a complexome analysis of *P. falciparum* [10,30]. Several of the subunits are predicted to have at least one transmembrane domain (TMD) (figure 3), making them candidates for being part of the missing membrane component of the SDH complex. At the outset of our study, none of the identified proteins had been validated as SDH subunits (neither in *T. gondii* nor in other organisms). However, the homologue of one of the proteins, *Tg*MPODD, was found to localize to the mitochondrion and be essential for transmission of *P. berghei* into its mosquito host [31]. We hypothesized that *Tg*MPODD was a myzozoan-specific subunit of SDH in *T. gondii*, and set out to characterize it.

**Figure 4.**
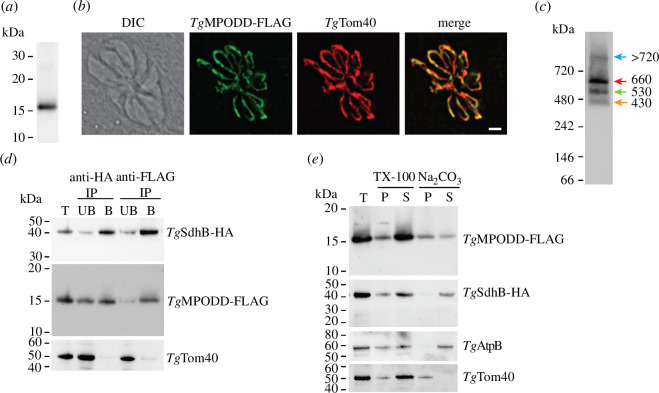
The myzozoan-specific protein *Tg*MPODD is an integral membrane component of the *T. gondii* SDH complex. (*a*) Western blots of proteins extracted from *Tg*MPODD-FLAG/*Tg*SdhB-HA parasites, separated by SDS–PAGE and probed using anti-FLAG antibodies. (*b*) Immunofluorescence assay of an eight-cell vacuole of *Tg*MPODD-FLAG/*Tg*SdhB-HA parasites, probed with anti-FLAG antibodies to detect *Tg*MPODD-FLAG (green) and anti-*Tg*Tom40 antibodies to detect the mitochondrion (red), with the overlap shown in the merged image. DIC, differential interference contrast transmission image. Scale bar is 2 µm. (*c*) Western blot of proteins extracted from *Tg*MPODD-FLAG/*Tg*SdhB-HA parasites in 1% (w/v) digitonin, separated by BN-PAGE, and detected with anti-FLAG antibodies. Masses of the observed complexes indicated with different coloured arrows next to bands were determined using relative migration distance (Rf) plots, taking the means of two independent experiments. (*d*) Immunoprecipitation (IP) of proteins extracted from *Tg*MPODD-FLAG/*Tg*SdhB-HA parasites in 1% (w/v) digitonin and incubated with either anti-FLAG or anti-HA agarose beads. The total fraction (T) was obtained from parasite lysates prior to immunoprecipitation. The unbound fractions (UB) contain proteins that were not retained on the agarose beads, and the bound fractions (B) contain proteins that were retained on the agarose beads. Fractions were separated by SDS–PAGE and probed with anti-HA antibodies to detect *Tg*SdhB-HA (top), anti-FLAG antibodies to detect *Tg*MPODD-FLAG (middle) or anti-*Tg*Tom40 antibodies (bottom) as a control for proteins that are not part of the SDH complex. Western blots are from a single experiment and are representative of two independent experiments. (*e*) TX-100 and alkaline sodium carbonate (Na_2_CO_3_) extractions of *Tg*MPODD-FLAG/*Tg*SdhB-HA parasites. Total fractions (T) were from proteins harvested before solubilization, and pellet (P) and soluble (S) fractions were harvested after each extraction. Proteins were separated by SDS–PAGE and probed with antibodies to detect *Tg*MPODD-FLAG (top), *Tg*SdhB-HA (second from top), the β subunit of ATP synthase, a known peripheral mitochondrial membrane protein (*Tg*AtpB; third from top) and *Tg*Tom40, a known integral membrane mitochondrial protein (bottom). Western blots are from a single experiment and are representative of two independent experiments.

To enable the detection of the *Tg*MPODD protein, we integrated a FLAG epitope tag at the 3′ end of the open reading frame of *Tg*MPODD in *Tg*SdhB-HA parasites, creating a line termed *T*gMPODD-FLAG/*Tg*SdhB-HA (electronic supplementary material, figure S3*a,b*). *Tg*MPODD-FLAG migrated to ~15 kDa when assessed by SDS–PAGE western blotting (figure 4*a*) and localized to the mitochondrion in immunofluorescence assays (figure 4*b*), consistent with the localization of its homologue in *P. berghei* [31]. To establish whether *Tg*MPODD is part of a protein complex, we extracted proteins from *Tg*MPODD-FLAG/*Tg*SdhB-HA parasites, separated them by BN-PAGE and performed western blotting to detect *Tg*MPODD-FLAG. We observed that *Tg*MPODD-FLAG is part of a main complex of ~660 kDa, and two less abundant complexes at ~530 and >720 kDa (figure 4*c*, magenta, green and blue arrows, respectively), similar to the masses of the *Tg*SdhB-containing complexes (figure 1*c*). Additionally, we found that *Tg*MPODD was part of a fourth complex at ~430 kDa (figure 4*c*, orange arrow). As a direct test of whether *Tg*MPODD is in the same complex as *Tg*SdhB, we solubilized proteins from *T*gMPODD-FLAG/*Tg*SdhB-HA parasites in 1% (w/v) digitonin and performed co-immunoprecipitation experiments. We found that immunoprecipitation of *Tg*SdhB-HA with anti-HA antibodies partially co-purified *Tg*MPODD-FLAG but not the unrelated mitochondrial protein *Tg*Tom40 (figure 4*d*). Similarly, we found that immunoprecipitation of *Tg*MPODD-FLAG with anti-FLAG antibodies co-purified *Tg*SdhB-HA but not *Tg*Tom40 (figure 4*d*). These data indicate that *Tg*MPODD is a component of the same protein complex(es) as *Tg*SdhB. While immunoprecipitation of *Tg*MPODD-FLAG resulted in co-purification of most of the *Tg*SdhB-HA protein, immunoprecipitation of *Tg*SdhB-HA co-purified proportionally less of the *Tg*MPODD protein (figure 4*d*). Together with the BN-PAGE data, these experiments are consistent with the hypothesis that *Tg*MPODD and *Tg*SdhB are both components of the major ~660 kDa SDH complex in addition to the two less prominent complexes of ~530 and >720 kDa and that *Tg*MPODD is a component of a fourth ~430 kDa complex from which *Tg*SdhB is absent.

The finding that *Tg*MPODD is a component of the SDH complex raises the possibility that it could contribute to the anchoring of this complex into the mitochondrial inner membrane. Previous bioinformatic analysis of the *P. berghei* homologue of MPODD predicted the presence of a single TMD in the protein [31], although our analysis of the *Tg*MPODD protein with some TMD prediction algorithms (including CCTOP [39]) does not predict the presence of a TMD figure 3. We therefore set out to experimentally test whether *Tg*MPODD is an integral membrane protein. To do this, we extracted proteins from *T*gMPODD-FLAG/*Tg*SdhB-HA parasites in either 1% (v/v) Triton X-100 or in alkaline sodium carbonate, an approach that extracts non-membrane and peripheral membrane proteins into the soluble fraction while maintaining integral proteins in the membrane pellet [42]. We found that the bulk of both *Tg*MPODD-FLAG and *Tg*SdhB-HA were soluble in Triton X-100, indicating that these proteins are not inherently insoluble (figure 4*e*). Notably, we found that most of the *Tg*MPODD-FLAG protein was retained in the alkaline sodium carbonate pellet along with the known integral membrane protein *Tg*Tom40, whereas both *Tg*SdhB-HA and the known peripheral inner mitochondrial membrane protein *Tg*ATPβ were fully extracted into the sodium carbonate supernatant (figure 4*e*). These data indicate that *Tg*MPODD-FLAG is an integral membrane protein, whereas *Tg*SdhB-HA is not. The existence of a proportion of *Tg*MPODD-FLAG in the sodium carbonate supernatant suggests that the TMD of *Tg*MPODD may be only mildly hydrophobic, as has been observed in proteins from other organisms [43] and as we have observed previously for other inner mitochondrial membrane proteins in *T. gondii* [44].

### 2.4. *Tg*MPODD is essential for SDH activity in *T. gondii*

**Figure 5.**
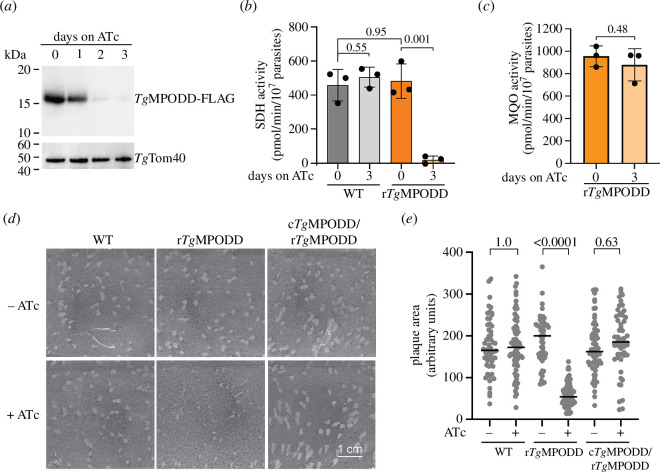
TgMPODD is critical for SDH activity and important for *T. gondii* proliferation. (*a*) Western blot of proteins extracted from r*Tg*MPODD-FLAG/*Tg*SdhB-HA parasites grown in the absence of ATc, or in the presence of ATc for 1–3 days, separated by SDS–PAGE and probed with anti-FLAG antibodies (to detect *Tg*MPODD-FLAG; top) and anti-*Tg*Tom40 antibodies (loading control; bottom). (*b*) SDH enzyme activity in WT parasites (grey, *Tg*MPODD-FLAG/*Tg*SdhB-HA parental line) and r*Tg*MPODD parasites (orange), cultured for 0 or 3 days in ATc. Columns represent mean ± s.d. of three independent experiments (individual points shown). ANOVA followed by Tukey’s multiple pairwise comparisons test was performed, with relevant *p*-values shown. (*c*) MQO enzyme activity in r*Tg*MPODD parasites cultured for 0 or 3 days in ATc. Columns represent mean ± s.d. of three independent experiments (individual points shown). An unpaired two-tailed *t*‐test was performed comparing the two conditions, with the *p*-value shown. (*d*) Plaque assays with WT parasites (parental line, *Tg*MPODD-FLAG/*Tg*SdhB-HA), r*Tg*MPODD parasites, and complemented c*Tg*MPODD/r*Tg*MPODD parasites, cultured in the absence (top) or presence (bottom) of ATc for 7 days. Images are from a single experiment and representative of three independent experiments. (*e*) Quantification of plaque sizes from the experiment shown in (*d*). Black lines depict the median plaque size (in arbitrary units of area) for each parasite line and condition. A one-way ANOVA followed by Tukey’s multiple pairwise comparisons test was performed, with relevant *p*-values shown.

Having established that *Tg*MPODD is a transmembrane subunit of the *T. gondii* SDH complex, we next wanted to investigate its role and importance in the complex. We replaced the native promoter of the *Tg*MPODD gene with an ATc-regulatable promoter in the *T*gMPODD-FLAG/*Tg*SdhB-HA line, creating a parasite line termed ‘r*Tg*MPODD’ (electronic supplementary material, figure S3*c,d*). To determine the extent of *Tg*MPODD knockdown, we cultured parasites in the absence of ATc or presence of ATc for 1–3 days, separated proteins by SDS–PAGE and measured *T*gMPODD-FLAG abundance by western blotting. We found that *Tg*MPODD-FLAG was mostly depleted after 2 days on ATc (figure 5*a*). To test whether *Tg*MPODD is important for SDH activity, we grew r*Tg*MPODD parasites with or without ATc for 3 days and measured SDH activity via enzymatic assays. SDH activity decreased by 96% when *Tg*MPODD was knocked down by the addition of ATc in the r*Tg*MPODD line (figure 5*b*). In contrast, SDH activity of the parental line (WT, *Tg*MPODD-FLAG/*Tg*SdhB-HA) was not affected by the addition of ATc (figure 5*b*), and we observed no significant change in MQO activity upon *Tg*MPODD knockdown (figure 5*c*). These data imply that depletion of *Tg*MPODD leads to a specific loss of SDH activity that mirrors the loss in activity we observed upon *Tg*SdhB depletion (figure 1*e*). Given that loss of *Tg*SdhB led to defects in ETC activity (figure 2*e*), it is likely that loss of *Tg*MPODD will lead to a similar depletion in ETC activity, although this is not something we tested.

Finally, we tested whether *Tg*MPODD is important for parasite proliferation. Depletion of *Tg*MPODD resulted in a proliferation defect that was rescued by complementing r*Tg*MPODD-FLAG/*Tg*SdhB-HA parasites with a constitutively expressed copy of *Tg*MPODD (figure 5*d,e*). The significant but partial proliferation defect observed when *Tg*MPODD was depleted resembles the proliferation defect observed when depleting or knocking out *Tg*SdhB (figure 2*a–c*). We conclude that *Tg*MPODD is crucial for SDH function in *T. gondii* parasites and that it is important but not essential for parasite proliferation.

### 2.5. *Tg*MPODD is important for the attachment of the catalytic subunits of the SDH complex to the integral membrane anchor

The SDH complex of *T. gondii* and related organisms appears to contain numerous novel subunits. To begin to elucidate the architecture of the SDH complex in *T. gondii*, we aimed to investigate how the loss of the matrix-localized *Tg*SdhB protein affects *Tg*MPODD and other putative subunits of the complex. We introduced c-myc epitope-tags into the genomic locus of *Tg*MPODD, as well as the loci of three additional subunits identified in the complexome analysis of the *T. gondii* SDH complex [12], *Tg*Sdh11, *Tg*Sdh15 and *Tg*Sdh31, in the r*Tg*SdhB-HA/*Tg*Sdh18-FLAG line (electronic supplementary material, figure S4). We validated that the previously uncharacterized candidate SDH proteins all localized to the mitochondrion of *T. gondii* (electronic supplementary material, figure S5*a–d*).

**Figure 6.**
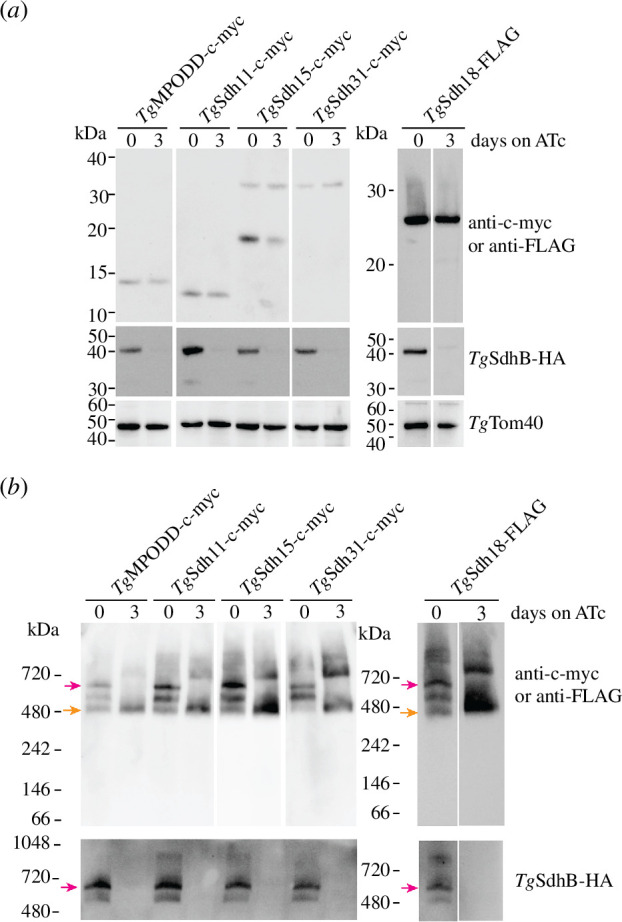
Depletion of *Tg*SdhB enhances the formation of a ~430 kDa subcomplex containing the candidate membrane-anchoring subunits of SDH. (*a*,*b*) Western blots of proteins extracted from *Tg*MPODD-c-myc, *Tg*Sdh11-c-myc, *Tg*Sdh15-c-myc or *Tg*Sdh31-c-myc expressing parasites, all tagged in an r*Tg*SdhB-HA/*Tg*Sdh18-FLAG background line, cultured in the absence of ATc or presence of ATc for 3 days. Proteins were separated by (*a*) SDS–PAGE or (*b*) BN-PAGE and detected with anti-c-myc, anti-FLAG, anti-HA or anti-*Tg*Tom40 antibodies. Western blots are from a single experiment and are representative of at least two independent experiments. For the BN-PAGE experiment, proteins were extracted in 1% (w/v) digitonin. The position of the ~660 kDa intact SDH complex and the putative ~430 kDa membrane anchoring subcomplex are indicated by magenta and orange arrows, respectively. Note that the day 0 and day 3 conditions on the *Tg*Sdh18-FLAG western blots are from the same exposure on the same membrane in an experiment where we also obtained data for parasites cultured for 1 and 2 days on ATc (electronic supplementary material, figure S6).

We first asked whether the abundances of the candidate SDH complex proteins changed upon depletion of *Tg*SdhB. We cultured parasites in the absence or presence of ATc for 3 days, separated proteins by SDS–PAGE, and measured protein abundances by western blotting. *Tg*MPODD-c-myc, *Tg*Sdh11-c-myc, *Tg*Sdh15-c-myc, *Tg*Sdh18-FLAG and *Tg*Sdh31-c-myc proteins were observed at approximately their predicted masses (figure 6*a*; electronic supplementary material, figure S6*a*). *Tg*Sdh15-c-myc was also observed in a second band of ~32 kDa, roughly double its expected mass, which could represent a dimer (figure 6*a*). Abundances of the *Tg*MPODD-c-myc, *Tg*Sdh11-c-myc, *Tg*Sdh18-FLAG and *Tg*Sdh31-c-myc proteins did not change appreciably upon the knockdown of *Tg*SdhB, while abundance of the lower mass band of *Tg*Sdh15-c-myc appeared to decrease (figure 6*a*; electronic supplementary material, figure S6*a*).

We next performed BN-PAGE on the tagged parasite strains cultured in the absence of ATc to test whether *Tg*Sdh11-c-myc, *Tg*Sdh15-c-myc, *Tg*Sdh18-FLAG and *Tg*Sdh31-c-myc existed in protein complexes. We found that, as observed previously (figure 4*c*), *Tg*MPODD-c-myc was present in a major complex of ~660 kDa (figure 6*b*, magenta arrow), in addition to two smaller complexes of ~530 and ~430 kDa (figure 6*b*). *Tg*Sdh11-c-myc, *Tg*Sdh15-c-myc and *Tg*Sdh18-FLAG were present in complexes of approximately the same masses as *Tg*MPODD-c-myc (figure 6*b* and electronic supplementary material, figure S6*b*). *Tg*Sdh31-c-myc was present in the ~660 and ~530 kDa complexes, but less clearly in the smaller ~430 kDa complex observed with the other proteins (figure 6*b*). Strikingly, when *Tg*SdhB was knocked down by the addition of ATc for 3 days, the ~660 kDa complex observed in the −ATc condition for all five subunits was absent. Instead, each protein migrated in a complex at ~430 kDa (figure 6*b*, orange arrow; electronic supplementary material, figure S6*b*) and also in a less abundant complex of >720 kDa (figure 6*b*). Taken together with our previous BN-PAGE analysis of *Tg*SdhB and *Tg*MPODD (figures 1*c* and 4*c*), these data imply that *Tg*Sdh11-c-myc, *Tg*Sdh15-c-myc, *Tg*Sdh18-FLAG and *Tg*Sdh31-c-myc are likely components of the ~660 kDa SDH complex. In addition, *Tg*Sdh11-c-myc, *Tg*Sdh15-c-myc and *Tg*Sdh18-FLAG are, together with *Tg*MPODD, all part of a smaller ~430 kDa complex from which *Tg*SdhB is absent. Given that *Tg*MPODD, *Tg*Sdh11 and *Tg*Sdh15 are integral membrane proteins (figure 4*e*) [45] and that *Tg*Sdh18 is predicted to contain TMDs (figure 3), we hypothesize that this smaller complex represents the membrane-anchoring domain of the SDH complex in these parasites.

**Figure 7.**
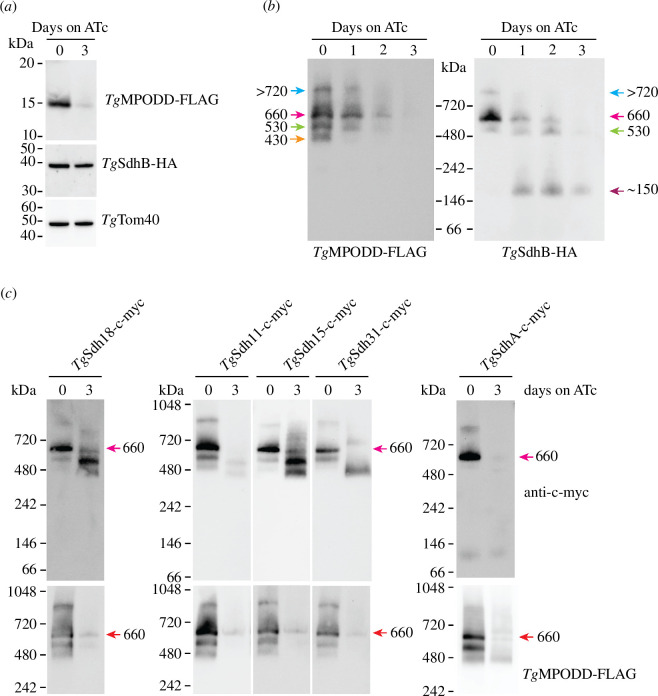
Loss of *Tg*MPODD causes dissociation of the matrix-localized catalytic subunits from the membrane anchor of the SDH complex. (*a*,*b*) Western blot of proteins extracted from r*Tg*MPODD-FLAG/*Tg*SdhB-HA parasites grown in the absence or presence of ATc for 3 days, separated by (*a*) SDS–PAGE or (*b*) BN-PAGE and detected using anti-FLAG, anti-HA or anti-*Tg*Tom40 antibodies as indicated. For BN-PAGE, proteins were extracted in 1% (w/v) digitonin buffer. Western blots are from a single experiment and are representative of two independent experiments. Masses indicated with different coloured arrows next to bands were determined via relative migration distance (Rf) plots, taking the means of two independent experiments. (*c*) Western blot of proteins extracted from *Tg*Sdh18-c-myc, *Tg*Sdh11-c-myc, *Tg*Sdh15-c-myc, *Tg*Sdh31-c-myc and *Tg*SdhA-c-myc expressing parasites, all tagged in a r*Tg*MPODD-FLAG/*Tg*SdhB-HA background, cultured in the absence or presence of ATc for 3 days. Proteins were extracted in 1% (w/v) digitonin, separated by BN-PAGE and detected with anti-c-myc or anti-FLAG antibodies as indicated. Western blots are from a single experiment and are representative of at least two independent experiments. The position of the ~660 kDa SDH complex is indicated with a magenta arrow.

To further explore SDH complex architecture, we investigated the importance of *Tg*MPODD for complex integrity. We cultured r*Tg*MPODD-FLAG/*Tg*SdhB-HA parasites in the absence or presence of ATc for 3 days and measured protein abundance by SDS–PAGE western blotting. We found that the abundance of *Tg*SdhB-HA does not change upon *Tg*MPODD depletion (figure 7*a*). We then performed BN-PAGE western blotting on proteins extracted from r*Tg*MPODD-FLAG/*Tg*SdhB-HA parasites cultured for 0–3 days on ATc. As observed previously, we found that in the absence of ATc, both *Tg*MPODD-FLAG and *Tg*SdhB-HA were present in a major complex of ~660 kDa, in addition to less abundant complexes at ~530 and >720 kDa (figure 7*b*, magenta, green and blue arrows, respectively). *Tg*MPODD-FLAG was additionally present in the ~430 kDa complex (figure 7*b*, orange arrow). Upon *Tg*MPODD-FLAG knockdown, we observed disappearance of the 430 kDa complex after 1 day on ATc and depletion of the other *Tg*MPODD-FLAG-containing complexes to undetectable levels after 3 days (figure 7*b*). Notably, the major 660 kDa *Tg*SdhB-HA-containing complex depleted concomitantly with depletion of the equivalent 660 kDa *Tg*MPODD-FLAG complex, with *Tg*SdhB-HA appearing in a new, smaller complex of ~150 kDa upon *Tg*MPODD-FLAG knockdown (figure 7*b*, purple arrow). These data indicate that depletion of *Tg*MPODD results in the loss of *Tg*SdhB from the SDH complex.

Having established that loss of *Tg*MPODD affects SDH complex integrity and *Tg*SdhB integration into this complex, we investigated what happens to the *Tg*Sdh11-, *Tg*Sdh15-, *Tg*Sdh18- and *Tg*Sdh31-containing complexes upon *Tg*MPODD knockdown. We introduced c-myc tags into the loci of *Tg*Sdh11, *Tg*Sdh15, *Tg*Sdh18 or *Tg*Sdh31 in the r*Tg*MPODD-FLAG/*Tg*SdhB-HA line (electronic supplementary material, figure S7*a–h*). We cultured parasites in the absence or presence of ATc for 3 days and then performed BN-PAGE western blotting on extracted proteins. As expected, all four c-myc-tagged proteins localized predominantly to the ~660 kDa complex in the −ATc condition, when parasites expressed *Tg*MPODD-FLAG (figure 7*c*, magenta arrow). Upon *Tg*MPODD-FLAG knockdown by the addition of ATc, this 660 kDa complex was depleted in all lines (figure 7*c*). In the *Tg*MPODD-FLAG knockdown condition, both *Tg*Sdh15-c-myc and *Tg*Sdh18-c-myc migrated in a major complex of ~500 kDa and a less abundant complex of ~400 kDa (figure 7*c*). *Tg*Sdh31-c-myc migrated in a major complex of ~400 kDa, whereas the *Tg*Sdh11-c-myc protein appeared to be depleted from all protein complexes (figure 7*c*). These data indicate that knockdown of *Tg*MPODD leads to defects in the integrity of the major ~660 kDa SDH complex, with the concomitant formation of smaller complexes containing some of the other SDH subunits.

In SDH complexes from other organisms, the SdhB protein links the matrix-localized, catalytic SdhA flavoprotein to the membrane anchoring SdhC and SdhD proteins [46]. Our finding that depletion of *Tg*MPODD resulted in a detachment of the *Tg*SdhB protein from other components of the SDH complex prompted us to examine the effects of *Tg*MPODD depletion on *Tg*SdhA. To test this, we introduced a c-myc-tag into the *Tg*SdhA locus in the r*Tg*MPODD-FLAG/*Tg*SdhB-HA parasite line (electronic supplementary material, figure S7*i,j*) and demonstrated that *Tg*SdhA-c-myc localized to the mitochondrion (electronic supplementary material, figure S5*e*). We cultured the resultant line in the absence or presence of ATc for 3 days, then separated protein complexes via BN-PAGE and probed for *Tg*SdhA-c-myc. When *Tg*MPODD was present, *Tg*SdhA was detected in a major complex of ~660 kDa (figure 7*c*), consistent with this complex being the catalytically active SDH complex. Upon *Tg*MPODD knockdown, the *Tg*SdhA-c-myc was not detected at any higher molecular mass complex (figure 7*c*), suggesting that it is entirely lost from the SDH complex.

Taken together, these data indicate that the loss of *Tg*MPODD causes major defects in the integrity of the SDH complex, with the matrix-localized catalytic *Tg*SdhA and *Tg*SdhB subunits dissociating from the complex entirely, and many of the other components (including *Tg*Sdh15, *Tg*Sdh18 and *Tg*Sdh31) forming smaller subcomplexes.

## 3. Discussion

In this study, we explored the physiological importance of the SDH complex in the disease-causing tachyzoite stage of the apicomplexan parasite *T. gondii*. We found that loss of *Tg*SdhB, the catalytically essential Fe–S component of the complex*,* resulted in reduced mOCR and parasite proliferation. A parallel study by Silva *et al*. reached similar conclusions about other subunits of the *T. gondii* SDH complex [45]. Although substantial, the observed defects in mOCR and proliferation upon *Tg*SdhB knockdown were milder than defects observed upon knockdown of *Tg*QCR11, an essential component of ETC complex III [11]. These data suggest that the parasite ETC remains functional in the absence of the SDH complex, with electrons likely donated to coenzyme Q via other inner membrane dehydrogenases that are found in the parasite mitochondrion (figure 2*d*) [1,47,48]. In addition to its role in the ETC, SDH catalyses a key reaction in the TCA cycle. Our data therefore imply that the TCA cycle is important but not absolutely essential for parasite proliferation, at least in the nutrient rich conditions in which we culture parasites *in vitro*. These data are somewhat surprising, given that treatment of parasites with sodium fluoroacetate, an inhibitor of the TCA cycle enzyme aconitase, fully inhibits tachyzoite proliferation [18,49]. A previous study of the succinyl-CoA synthetase enzyme in *T. gondii*, which catalyses the synthesis of succinate in the TCA cycle, found that knockdown of the ScsA protein from this enzyme complex led to only a mild proliferation defect [22]. The dispensability of succinyl-CoA synthetase was proposed to result from the existence of a GABA shunt, which bypasses the α-ketoglutarate dehydrogenase- and succinyl-CoA synthetase-catalysed reactions of the TCA cycle [18]. The SDH-catalysed reaction, however, occurs downstream of the proposed GABA shunt, suggesting that its dispensability cannot be explained in this manner. Future experiments that assess changes to parasite metabolism associated with SDH loss and a broader characterization of the essentiality of TCA cycle enzymes will be key to resolving the role and importance of the TCA cycle in *T. gondii* tachyzoites.

Our findings on the physiological role of the SDH complex fit with a broader narrative that *T. gondii* tachyzoites exhibit considerable metabolic flexibility that may contribute to their ability to infect a broad range of host organisms and tissues [50,51]. Future studies that examine the effects of SDH and TCA cycle impairment on the ability of parasites to cause disease in whole animal infection models will be crucial for understanding the role of mitochondrial energy metabolism in this proposed flexibility. The SDH complex, and in particular, the membrane anchor of the complex, is a major target of pesticides that target fungal pathogens in plants [52]. Given the novelty in protein composition of the SDH complex in apicomplexans, particularly in the membrane anchoring subunits, the SDH complex is an attractive target for inhibitors. Elucidating the contribution of the SDH complex for parasite virulence is therefore also of high importance for determining whether the SDH complex could be developed as a drug target in these parasites.

A bevy of recent studies has established that the mitochondrial ETC complexes of myzozoans, including *T. gondii* and other apicomplexans, contain numerous novel and divergent subunits compared to the equivalent protein complexes in well-studied organisms such as fungi and animals [9–12,44,45]. Given that the common ancestor of myzozoans and opisthokonts (fungi and animals) probably existed at the dawn of eukaryotic evolution [53], this suggests that considerable novelty in ETC protein complexes arose over the course of myzozoan evolution. A major focus is now on elucidating the functional roles of the novel proteins in these complexes [1,44]. Building on two recent ‘complexome’ studies [10,12], our data establish that MPODD is a *bona fide* component of the SDH complex of *T. gondii*. A previous study established that MPODD is a myzozoan-specific protein that is essential for development of ookinetes in the early insect stages of *Plasmodium* parasite development [31]. Other studies have established that the SDH complex and other proteins of the TCA cycle are important for early insect stage development in *Plasmodium* parasites, possibly because of an increased reliance on oxidative phosphorylation or products of TCA cycle metabolism in these stages [20,32]. Our findings that MPODD is essential for SDH function in these parasites provide a rationale for its importance in ookinete development.

**Figure 8.**
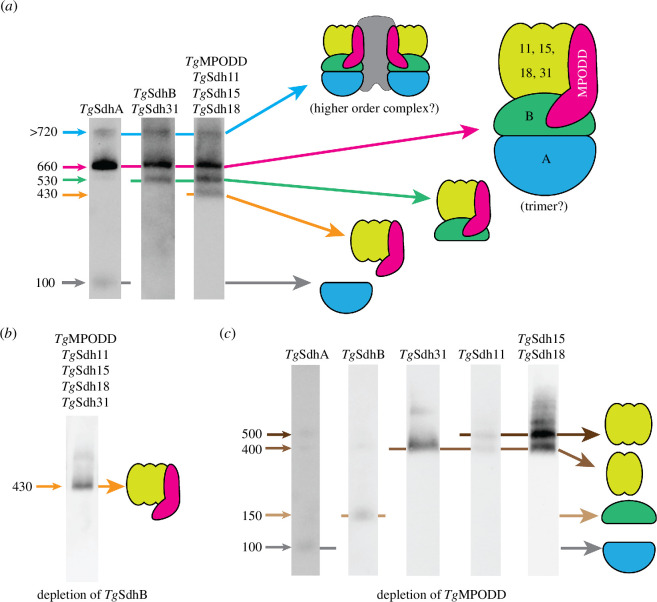
A model for the architecture of the SDH complex in *T. gondii*. (*a*) The functional *T. gondii* SDH complex comprises a ~660 kDa complex that contains all proposed subunits of the complex and may exist as a trimer (magenta arrow). The complex also exists in a higher-order structure of >720 kDa (blue arrow). Smaller complexes observed represent the membrane anchor lacking *Tg*SdhA and with the *Tg*SdhB protein tethered (~530 kDa complex; green arrow) or not-tethered (~430 kDa complex; orange arrow). (*b*) Depletion of *Tg*SdhB leads to the formation of a ~430 kDa complex that contains the membrane-bound subunits of the complex. We propose that this represents the membrane anchor of the SDH complex in *T. gondii*. (*c*) Depletion of *Tg*MPODD leads to the loss of *Tg*SdhA and *Tg*SdhB from the complex (grey and light brown arrows), with remnant complexes of ~400 and ~500 kDa containing some of the membrane anchoring subunits of the complex (dark brown arrows). These data suggest a role for *Tg*MPODD in tethering the matrix components *Tg*SdhB and *Tg*SdhA to the membrane anchoring subcomplex. BN-PAGE data in the figure are derived from experiments depicted in figures 1, 3, 5 and 6.

When solubilized in the mild detergent digitonin, the SDH complex exists primarily in a ~660 kDa complex. We propose that this complex represents the functional unit of the SDH complex in parasites, since it contains the matrix-localized SdhA and SdhB that are essential for the catalytic activity of the complex (figure 8*a*). Although the stoichiometry of proteins within the complex remains unclear, the sum of the SDH subunits adds to ~230 kDa (figure 3). This suggests that the 660 kDa complex may represent a trimer, as has been proposed for the equivalent complex in *P. falciparum* parasites [10]. We also observe a large complex of >720 kDa that could either represent a larger order arrangement of SDH complexes or an ETC supercomplex, an arrangement of multiple ETC complexes in a single larger complex that has been observed in many other eukaryotes [54] (figure 8*a*). Although the existence of SDH in such supercomplexes is not as common [55], proteomic analysis of a putative ~745 kDa SDH complex in the related parasite *Eimeria tenella* identified complex III and IV proteins [56], and the SDH complex of the ciliate *Tetrahymena* (a sister taxon to the myzozoans) exists in a massive ETC supercomplex [57]. Curiously, our BN-PAGE experiments on numerous SDH subunits revealed the presence of subcomplexes of ~530 and ~430 kDa. These smaller complexes contained membrane-bound proteins such as *Tg*MPODD, *Tg*Sdh15 and *Tg*Sdh18 but lacked the soluble catalytic *Tg*SdhA in the 530 kDa complex and the *Tg*SdhA and *Tg*SdhB subunits in the 430 kDa complex (figure 8*a*). Following *Tg*SdhB depletion, a ~430 kDa complex containing the proposed membrane-bound subunits remains (figure 8*b*), and we propose that this complex represents the membrane anchor to which *Tg*SdhB and *Tg*SdhA attach to form the catalytically active SDH complex, although it is also conceivable that this represents an assembly intermediate. Interestingly, the 430 kDa complex was not observed in the Silva *et al*. study, which used *n*-dodecyl-β-d-maltoside (DDM) to solubilize proteins for BN-PAGE analysis [45]. DDM is a ‘harsher’ detergent than the digitonin we used in our study, which may reflect that the proposed 430 kDa membrane anchor is particularly labile. Future structural studies of the various SDH complexes in the parasite may provide insights into the nature and function of these subcomplexes.

Our experiments reveal that *Tg*MPODD is an integral membrane protein that is essential for SDH complex function. What then is the role of *Tg*MPODD in the complex? Strikingly, depletion of MPODD results in loss of the catalytic *Tg*SdhB and *Tg*SdhA subunits from the SDH complex and the formation of smaller complexes of ~400 and ~500 kDa that contain many of the proposed membrane anchoring subunits (figure 8*c*). We propose that *Tg*MPODD plays a crucial role in recruiting and/or tethering the *Tg*SdhB and *Tg*SdhA subunits to the membrane anchoring proteins of the complex. Given that the channelling of electrons from the Fe–S clusters of *Tg*SdhB to ubiquinone at the membrane anchor is critical for SDH function, this model explains the essentiality of *Tg*MPODD for SDH activity. Future structural studies that elaborate on the position of *Tg*MPODD in the SDH complex of apicomplexans and related organisms may provide insights into how this recruitment or tethering is mediated.

Our study explored the role and architecture of the SDH complex in *T. gondii,* demonstrating that *Tg*MPODD is an essential, membrane-anchored subunit of the SDH complex, with an important role in maintaining complex integrity and tethering the matrix-localized SdhA and SdhB to the membrane anchor. Our data therefore provide insights into the previously unknown function of this protein. Future studies should examine whether the functions of MPODD holds true in other myzozoans, and further elucidate the structural architecture of the SDH complex in these organisms.

## 4. Material and methods

### 4.1. Host cell and parasite culture

*Toxoplasma gondii* tachyzoites were cultured in human foreskin fibroblast host cells in Dulbecco’s modified Eagle’s medium containing 2 g  l^−1^ NaHCO_3_, supplemented with 1% (volume by volume (v/v)) foetal bovine serum, 50 U ml^−1^ penicillin, 50 µg ml^−1^ streptomycin, 10 µg ml^−1^ gentamicin, 0.25 µg ml^−1^ amphotericin B and an additional 0.2 mM l-glutamine. Parasite cultures were kept in a humidified 37°C incubator at 5% CO_2_. Where applicable, anhydrotetracycline (ATc) was added to a final concentration of 0.5 μg ml^−1^ and was paired with an ethanol vehicle control (0.025% v/v).

### 4.2. Genetic modifications of *T. gondii*

Most of the genetically modified parasite lines described in the study were generated in the TATi∆*ku80* strain of *T. gondii* [58] and were cloned before subsequent characterization. All transfections and drug selections were performed as described previously [59].

We introduced a 3′ haemagglutinin (HA) into the *Tg*SdhB locus of the TATi∆*ku80* strain using a selectable marker-based 3′ replacement strategy described previously [34], generating a strain we termed *Tg*SdhB-HA. We next introduced FLAG tags into the *Tg*Sdh18 or *Tg*MPODD loci of this strain using a selection marker-less CRISPR/Cas9 genome editing approach described previously [9]. To do this, we generated a vector expressing a single guide (sg)RNA targeting the region around the predicted stop codon of *Tg*Sdh18 or *Tg*MPODD by modifying the pSAG1::Cas9-U6::sgUPRT vector (Addgene plasmid, 54467 [60]) using Q5 mutagenesis with the *Tg*Sdh18 3′ CRISPR fwd or *Tg*MPODD 3′ CRISPR fwd primers together with the universal CRISPR rvs primer (electronic supplementary material, table S1) as per the manufacturer’s instructions. We then PCR amplified a FLAG tag with 50 bp homology arms to the *Tg*Sdh18 or *Tg*MPODD loci at either end using the primers *Tg*Sdh18 tag fwd and rvs or *Tg*MPODD tag fwd and rvs and a FLAG gBlock (Integrated DNA Technologies, (IDT)) as template (electronic supplementary material, table S1). We co-transfected the resulted PCR product and sgRNA-expressing plasmid into *Tg*SdhB-HA parasites, selected and cloned GFP-positive parasites (which express the Cas9-GFP encoded on the sgRNA-expressing vector) by fluorescence-activated cell sorting (FACS) using a FACSMelody cell sorter (BD Biosciences) 2 or 3 days after transfection, then screened for parasites that had incorporated the FLAG tag into the *Tg*Sdh18 or *Tg*MPODD loci using the *Tg*Sdh18 or *Tg*MPODD 3′ scrn fwd and rvs primers (electronic supplementary material, table S1). This generated strains we termed *Tg*SdhB-HA/*Tg*Sdh18-FLAG or *Tg*SdhB-HA/*Tg*MPODD-FLAG.

Next, we introduced an ATc-regulatable promoter upstream of the *Tg*SdhB or *Tg*MPODD start codons of the *Tg*SdhB-HA/*Tg*Sdh18-FLAG or *Tg*SdhB-HA/*Tg*MPODD-FLAG lines, respectively, using a selection marker-less CRISPR/Cas9 genome editing-based promoter insertion strategy described previously [9]. We performed Q5 mutagenesis to modify the pSAG1::Cas9-U6::sgUPRT vector to express sgRNAs targeting the 5′ region of the *Tg*SdhB or *Tg*MPODD genes close to the start codon using the *Tg*SdhB 5′ CRISPR fwd or *Tg*MPODD 5′ CRISPR fwd primer together with the universal CRISPR rvs primer (electronic supplementary material, table S1). We also amplified the ATc-regulatable teto7/sag4 promoter [61] and an associated spacer region along with 50 bp homology arms targeting the *Tg*SdhB or *Tg*MPODD loci using the primers SdhB prorep (promoter replacement) fwd and rvs or MPODD prorep fwd and rvs (electronic supplementary material, table S1) and the pPR2-HA_3_ vector [62] as template. We co-transfected the PCR products and sgRNA-expressing plasmid into *Tg*SdhB-HA/*Tg*Sdh18-FLAG or *Tg*SdhB-HA/*Tg*MPODD-FLAG parasites, sorted and cloned parasites 2 or 3 days after transfection as described above and screened clones for integration of the ATc-regulatable promoter using the SdhB 5′ scrn fwd and rvs or MPODD 5′ scrn fwd and rvs primers (electronic supplementary material, table S1). We referred to the resulting ATc-regulatable (r) *Tg*SdhB-HA/*Tg*Sdh18-FLAG line as ‘r*Tg*SdhB’ throughout the manuscript and the resulting r*Tg*MPODD-FLAG/*Tg*SdhB-HA line as ‘r*Tg*MPODD’.

To complement the r*Tg*SdhB and r*Tg*MPODD lines with constitutively expressed, Ty1-tagged *Tg*SdhB and *Tg*MPODD, respectively, we PCR amplified the open reading frames of *Tg*SdhB or *Tg*MPODD using the primers SdhB comp fwd or MPODD comp fwd together with Ty1 universal rvs (electronic supplementary material, table S1), and using gBlocks encoding the entire open reading frames of either *Tg*SdhB or *Tg*MPODD fused to a 3 × Ty1 epitope tag as templates (electronic supplementary material, table S1; IDT). We digested the resulting PCR products with *Bgl*II and *Xma*I and ligated these into the equivalent sites of a vector termed *Tg*QCR11 in pUDT-Ty1 [11]. The resultant vector expresses the *Tg*SdhB-Ty1 or *Tg*MPODD-Ty1 transgenes from the constitutive α-tubulin promoter and contains a pyrimethamine-resistant *Tg*DHFR selectable marker and a UPRT flanking sequence for integration into the non-essential uracil phosphoribosyltransferase (*Tg*UPRT) locus of the parasite. The resultant *Tg*SdhB-Ty1 or *Tg*MPODD-Ty1 expressing vectors were linearized in their UPRT flank with *Mfe*I, transfected into r*Tg*SdhB or r*Tg*MPODD lines, respectively, selected on pyrimethamine and then cloned by limiting dilution. Although we anticipate that the vector should integrate into the UPRT locus of the parasite, we did not check for this.

To introduce c-myc tags into the *Tg*MPODD, *Tg*Sdh11, *Tg*Sdh15, *Tg*Sdh18, *Tg*Sdh31 or *Tg*SdhA loci of the r*Tg*SdhB and *Tg*MPODD lines, we used the same selection marker-less CRISPR/Cas9 genome editing approach described above. Briefly, we generated vectors expressing sgRNAs targeting near the predicted stop codons of the target genes using Q5 mutagenesis with the gene-specific ‘3′ CRISPR fwd’ primer and the universal CRISPR rvs primer (electronic supplementary material, table S1). We also amplified a 3 × c-myc epitope tag containing 50 bp homology arms to the target gene with the gene-specific ‘tag fwd’ and ‘tag rvs’ primers together with a c-myc gBlock (IDT) as template (electronic supplementary material, table S1). We co-transfected the sgRNA-expressing vector and 3 × c-myc homology template into r*Tg*SdhB or r*Tg*MPODD parasites, selected and cloned Cas9-GFP expressing parasite by flow cytometry 2 or 3 days after transfection and then screened for successful integration of the c-myc tag using the gene-specific ‘3′ scrn fwd’ and ‘3′ scrn rvs’ primers (electronic supplementary material, table S1).

To generate a parasite strain in which the *Tg*SdhB open reading frame was disrupted (i.e. where the *Tg*SdhB was functionally knocked out), we first modified the pSAG1::Cas9-U6::sgUPRT vector by Q5 mutagenesis to express a sgRNA targeting the open reading frame in the second exon of the *Tg*SdhB locus. We performed the Q5 mutagenesis using the SdhB KO CRISPR fwd and universal CRISPR rvs primers (electronic supplementary material, table S1), as per the manufacturer’s instructions. We also PCR amplified a phleomycin selectable marker (BLE) containing 50 bp homology arms to the *Tg*SdhB locus on either side of the sgRNA-targeting site with the primers SdhB KO fwd and rvs (electronic supplementary material, table S1) and the vector pUBTTy as template [63]. We co-transfected the sgRNA-expressing vector and BLE-containing homology template into the Cas9-CAT parasite line (a kind gift from Clare Harding, U. Glasgow [64]), selected and cloned Cas9-GFP-expressing parasite by flow cytometry 2 days after transfection and then screened for successful integration of DNA into the *Tg*SdhB open reading frame using the SdhB KO scrn fwd and rvs primers (electronic supplementary material, table S1).

### 4.3. Plaque assays

Plaque assays were performed as described previously [44], with 500 parasites added per flask. Flasks were incubated for 7 days before staining with crystal violet (Con and Hucker’s formula, Fronine Laboratory Supplies or Gram’s Crystal Violet solution, Sigma). Flasks were left to dry and scanned using a CanoScan 9000F scanner (Canon). Plaque areas were determined using the freehand area selection and measure tools in ImageJ (v. 1.53 k), with plaques that had merged with neighbouring plaques excluded from the analysis. Statistical comparisons of the mean plaque areas for each parasite line and condition was performed using a one-way ANOVA with Tukey’s multiple comparisons test in GraphPad Prism (v. 10).

### 4.4. Sodium dodecyl sulfate–polyacrylamide gel electrophoresis, blue native-PAGE and immunoblotting

Sodium dodecylsulfate (SDS)-polyacrylamide electrophoresis (PAGE), blue native (BN)-PAGE and immunoblotting were performed as described previously [65]. SDS–PAGE samples were solubilized in NuPAGE lithium dodecyl sulfate (LDS) sample buffer containing 2.5% (v/v) β-mercaptoethanol (reducing LDS sample buffer) and separated on a 12% (v/v) NuPAGE Bis–Tris polyacrylamide gel (Thermo Fisher Scientific). BN-PAGE samples were solubilized in NativePAGE sample buffer containing 1% weight by volume (w/v) digitonin and protease inhibitors and separated on a NativePAGE 4–16% (v/v) Bis–Tris polyacrylamide gel (Thermo Fisher Scientific). Primary antibodies used for western blotting included mouse anti-FLAG (1 : 500–1 : 3000 dilution; clone M2; Sigma, catalogue number F3165), rat anti-HA (1 : 1000 dilution; clone 3F10; Sigma, catalogue number 867423001), mouse monoclonal IgG1 anti-c-myc (1 : 250–1 : 500 dilution; clone 9E10; Santa Cruz Biotechnology, catalogue number SC-40) and rabbit anti-Tom40 (1 : 2000 dilution [65]). The secondary antibodies used were horseradish peroxidase (HRP)-conjugated goat anti-mouse IgG (Abcam, catalogue number ab6789), goat anti-rabbit IgG (Abcam, catalogue number ab97051) and goat anti-rat IgG (Abcam, catalogue number ab97057). Blots were imaged using a ChemiDoc MP imaging system (BioRad).

### 4.5. Sodium carbonate extraction assays

To test whether proteins were integral membrane proteins, we solubilized parasite fractions in alkaline sodium carbonate (Na_2_CO_3_, pH 12), as described previously [34,66]. Briefly, egressed parasites were resuspended in phosphate-buffered saline (PBS) and split into three equal volumes and pelleted at 12 000 × *g* for 1 min. One pellet was resuspended in 1× reducing LDS sample buffer to yield the ‘Total’ fraction. The second pellet was resuspended in TX-100 lysis buffer (1% v/v Triton X-100, 150 mM NaCl, 2 mM EDTA and 50 mM Tris (pH 7.4), with added complete protease inhibitors (Sigma)), incubated on ice for 30 min, then centrifuged at 21 000 × *g* for 20 min at 4°C. The resulting pellet, containing TX-100 insoluble protein, was resuspended in 1× reducing LDS sample buffer. The TX-100 soluble proteins in the supernatant were precipitated via trichloroacetic acid precipitation, as described previously [34], before being resuspended in 1× reducing LDS sample buffer. The third parasite fraction was resuspended in 100 mM of alkaline Na_2_CO_3_ (pH 12), incubated on ice for >60 min and then ultracentrifuged at 189 000 × *g* for 30 min at 4°C. The resulting pellet, containing integral membrane proteins, was resuspended in 1× reducing LDS sample buffer. The Na_2_CO_3_ supernatant, containing soluble proteins and peripheral membrane proteins, was TCA precipitated and resuspended in 1× reducing LDS sample buffer. Samples were separated by SDS–PAGE and proteins were detected by western blotting, as described above.

### 4.6. Immunofluorescence assays and microscopy

Immunofluorescence assays were performed as described previously [66]. Primary antibodies used were mouse anti-FLAG (1 : 500–1 : 3000 dilution; clone M2; Sigma, catalogue number F3165), rat anti-HA (1 : 100 dilution; clone 3F10; Sigma, catalogue number 867423001), mouse anti-c-myc (1 : 250–1 : 500 dilution; clone 9E10; Santa Cruz Biotechnology, catalogue number SC-40) and rabbit anti-Tom40 (1 : 2000 dilution [65]). Secondary antibodies used were goat anti-mouse Alexa Fluor 488 (1 : 500 dilution; Thermo Fisher Scientific, catalogue number A-11029), goat anti-rat Alexa Fluor 488 (1:500 dilution; Thermo Fisher Scientific, catalogue number A-11006) and goat anti-rabbit Alexa Fluor 546 (1 : 500 dilution; Thermo Fisher Scientific, catalogue number A-11035). Images were acquired on a DeltaVision Elite deconvolution microscope (GE Healthcare) fitted with a 100× UPlanSApo oil immersion objective lens (Numerical Aperture 1.40). Images were deconvolved and adjusted linearly for contrast and brightness using SoftWoRx Suite 2.0 software and subsequently processed using Adobe Illustrator.

### 4.7. Co-immunoprecipitation experiments

Immunoprecipitations were performed as described previously [65,66], with parasite proteins solubilized in lysis buffer (50 mM Tris–HCl (pH 8.0), 150 mM NaCl and 2 mM EDTA with complete protease inhibitors) containing 1% (w/v) digitonin. HA-tagged proteins were immunoprecipitated using anti-HA affinity matrix (Sigma, catalogue number 11815016001) and FLAG-tagged proteins were immunoprecipitated using anti-FLAG M2 affinity gel (Sigma, catalogue number A2220). Proteins were eluted from affinity beads by boiling in 1× reducing LDS sample buffer, separated by SDS–PAGE and probed by western blotting as described above. To detect mouse antibodies on immunoprecipitated samples, HRP-conjugated anti-mouse TrueBlot Ultra secondary antibodies (1:2500 dilution; Rockland Immunochemicals, catalogue number 18-18817-33) were used.

### 4.8. Complex II and MQO enzymatic activity assays

Enzymatic activity assays were adapted from Spinazzi *et al*. [67]. In these assays, parasite extracts were incubated in assay buffer containing the substrate succinate (to measure complex II activity) or malate (to measure MQO activity) and the redox dye 2,6-dichlorophenolindophenol (DCPIP). When oxidized, DCPIP is blue with a maximal absorbance at 600 nm; when reduced, DCPIP is colourless. The reaction is started by adding the ubiquinone analogue decylubiquinone (DUB). The parasite complex II or MQO, if functional, will oxidize succinate/malate and reduce DUB to DUBH_2_. DUBH_2_ then reduces the DCPIP, rendering it colourless. Enzyme activity will, therefore, result in a decrease in absorbance of the sample at 600 nm over time.

Egressed parasites were passed through a 5 µm polycarbonate filter to remove host cell debris, counted using a haemocytometer and pelleted by centrifugation (10 min, 1500 × *g,* room temperature (RT)). Pellets were washed in 1 ml cold PBS and centrifuged (1 min, 12 000 × *g,* RT). Parasites were resuspended to 2.5 × 10^8^ parasites/ml in mitochondrial assay buffer (70 mM sucrose, 220 mM mannitol, 10 mM KH_2_PO_4_, 5 mM MgCl_2_, 2 mM HEPES, 1 mM EGTA, 0.2% w/v fatty-acid-free BSA and 0.2% w/v digitonin, pH 7.2) and lysed on a spinning wheel (30 min, 4°C).

Enzyme assay buffer containing 20 mM succinate or malate, 25 mM KH_2_PO_4_, 0.002175% w/v DCPIP, 1 µM atovaquone and 2.5 mg ml^−1^ fatty-acid-free BSA was prepared and aliquoted into the wells of a 24-well plate. A baseline reading was taken by measuring the absorbance at 600 nm every 30 s for 2 min using a TECAN Infinite 200 PRO plate reader warmed to 37°C. Parasite lysate (an equivalent of 6.25 × 10^7^ parasites per ml) was then added to the wells and a further baseline reading was taken every 30 s for 15 min. To start the reaction, DUB was added to a final concentration of 5 µM, and DCPIP absorbance at 600 nm was measured every 30 s for 45 min.

To calculate enzymatic activity, absorbance was plotted as a function of time. The initial rate was estimated from the first 5 min after adding DUB (when the reaction was in linear phase) and divided by the extinction coefficient for DCPIP (19.1 mM cm^−1^) according to the Beer–Lambert law. For each condition, the background (initial rate in the absence of parasite lysate) was subtracted from the observed value to yield the calculated activity. As a statistical test for differences between conditions, we performed ANOVAs followed by multiple pairwise comparison testing or unpaired, two-tailed *t*-tests. Statistical analyses of the data were carried out in RStudio and GraphPad Prism. Graphs were built using GraphPad Prism.

### 4.9. Seahorse XFe96 extracellular flux analysis

Experiments measuring the mitochondrial oxygen consumption rate (mOCR) of intact extracellular parasites using a Seahorse XFe96 extracellular flux analyser were conducted as described previously [35]. Data from the Seahorse flux analysis were exported from the Seahorse Wave Desktop software (Agilent Technologies). A linear mixed-effects model was applied to the data as described previously [35], setting the error between plates (between experiments) and wells (within experiments) as random effects, and the mOCR values between cell lines and days on drug (ATc) as fixed effects. Analysis of the least square means of the values was performed in the R software environment. Statistical differences between these values were tested through ANOVA (linear mixed effects), with a *post hoc* Tukey test.

## Data Availability

All data are presented in the manuscript or electronic supplementary material [68].
